# Curcumin and Novel Synthetic Analogs in Cell-Based Studies of Alzheimer’s Disease

**DOI:** 10.3389/fphar.2018.01404

**Published:** 2018-12-03

**Authors:** Stella Gagliardi, Valentina Franco, Stefano Sorrentino, Susanna Zucca, Cecilia Pandini, Paola Rota, Stefano Bernuzzi, Alfredo Costa, Elena Sinforiani, Orietta Pansarasa, John R. Cashman, Cristina Cereda

**Affiliations:** ^1^Genomic and Post-Genomic Center, IRCCS Mondino Foundation, Pavia, Italy; ^2^Clinical and Experimental Pharmacology Unit, Department of Internal Medicine and Therapeutics, University of Pavia, Pavia, Italy; ^3^Department of Biology and Biotechnology “L. Spallanzani”, University of Pavia, Pavia, Italy; ^4^Laboratory of Stem Cells for Tissue Engineering, IRCCS Policlinico San Donato, Milan, Italy; ^5^Department of Biomedical, Surgical and Dental Sciences, University of Milan, Milan, Italy; ^6^Immunohematological and Transfusional Service, Centre of Transplantation Immunology, Fondazione IRCCS Policlinico San Matteo, Pavia, Italy; ^7^Department of Brain and Behavioral Sciences, University of Pavia, Pavia, Italy; ^8^Neurology Department, IRCCS Mondino Foundation, Pavia, Italy; ^9^Laboratory of Neuropsychology/UVA, IRCCS Mondino Foundation, Pavia, Italy; ^10^Laboratory of Chemistry, Human BioMolecular Research Institute, San Diego, CA, United States

**Keywords:** Alzheimer’s disease, curcumins, next generation sequencing, NF-κB, amyloid beta, mannosyl-glycoprotein 4-beta-N-acetylglucosaminyltransferase

## Abstract

Alzheimer’s disease (AD) is a chronic neurodegenerative disorder that is associated with the most common type of dementia and is characterized by the presence of deposits of the protein fragment amyloid beta (Aβ) in the brain. The natural product mixture of curcuminoids that improves certain defects in innate immune cells of AD patients may selectively enhance Aβ phagocytosis by alteration of gene transcription. In this work, we evaluated the protective effects of curcuminoids in cells from AD patients by investigating the effect on NF-κB and BACE1 signaling pathways. These results were compared to the gene expression profile of the clearance of Aβ. The minor curcumin constituent, bisdemethoxycurcumin (BDC) showed the most potent protective action to decrease levels of *NF-κB* and *BACE1*, decrease the inflammatory cascade and diminish Aβ aggregates in cells from AD patients. Moreover, mannosyl-glycoprotein 4-beta-N-acetylglucosaminyltransferase (*MGAT3*) and vitamin D receptor (*VDR*) gene mRNAs were up-regulated in peripheral blood mononuclear cells from AD patients treated with BDC. BDC treatment impacts both gene expression including Mannosyl (Beta-1,4-)-Glycoprotein Beta-1,4-N-Acetylglucosaminyltransferase, Vitamin D and Toll like receptor mRNA and Aβ phagocytosis. The observation of down-regulation of *BACE1* and *NF-κB* following administration of BDC to cells from AD patients as a model system may have utility in the treatment of asymptomatic AD patients.

## Introduction

Alzheimer’s disease (AD) is a chronic neurodegenerative disorder that represents the most common type of dementia. AD is characterized by the presence of deposits of the protein fragment amyloid beta (Aβ) and twisted fibers of the protein tau (tangles) in the brain (McKhann et al., 1984; Selkoe, 2001; Blennow et al., 2006). The disproportionate accumulation and aberrant aggregation of Aβ is associated with the onset of neurodegenerative processes. Medications available to treat AD address symptomatic issues and target glutamatergic and cholinergic neurotransmissions and often show only modest clinical benefits (Kumar et al., 2015). Recent achievements in clinical trials of novel disease-modifying drugs such as aducanumab, show that targeting Aβ clearance represents a promising strategy in prevention and treatment of AD (Panza et al., 2016). Aducanumab decreased Aβ accumulation and slows cognitive decline in subjects during early stages of AD or in mild cognitive impairment patients (Panza et al., 2016). In addition to drugs, the study of naturally occurring compounds may offer promise for a novel therapeutic approach to treat AD.

Curcumin [i.e., 1,7-bis(4-hydroxy-3-methoxyphenyl)-1,6-heptadiene-3,5-dione, Compound 3] is a polyphenolic compound derived from the dried rhizome of the plant Curcuma Longa (Anderson et al., 2000). In animal studies, curcumin has been shown to possess neuroprotection biological properties due to its anti-inflammatory and anti-oxidant effects (Aggarwal and Harikumar, 2009; Cashman et al., 2012). Curcumin was reported to promote *in vivo* disaggregation of existing Aβ deposits and avoid new aberrant Aβ aggregation into fibrils (Zhang et al., 2006). However, clinical utility of curcumin has been limited because of its poor systemic bioavailability and chemical instability. Ahmed et al. (2010) described the effects of intraperitoneally injected curcuminoid mixtures [curcumin, bisdemethoxycurcumin (BDC) and demethoxycurcumin] and individual components on memory enhancement in an amyloid-infused rat model. The study found that, compared to curcumin, BDC (Compound 1) and demethoxycurcumin exerted a more efficacious effect on memory enhancement.

It may be that regulation of transcription factors, cytokines and enzymes associated with NF-κB functional activity is responsible for the mechanism of action of natural curcumins and analogs to human cells of relevance to AD (Fiala et al., 2007). In particular, BDC appears to be the most efficient compound in enhancing macrophage-promoted phagocytosis and Aβ clearance (Fiala et al., 2007). BDC could have anti-inflammatory action related to the NF-κB transcription pathway by promoting over-expression of MGAT3 (*GnT-III*) and Toll-like Receptors (*TLRs*), both known to be essential for normal macrophage function usually decreased in subjects with AD (Fiala et al., 2007). In fact, it was amply shown that lipopolysaccharide (LPS) binds to TLR4, and leads to the activation of NF-κB signaling and to activation of macrophages (Taro and Akira, 2007). Moreover, the implication of NF-κB and TLR4 in innate immunity and its link to phagocytosis by MGAT3 was described previously (Fiala et al., 2010).

In our previous study (Gagliardi et al., 2012) synthetic curcumin derivatives and other molecules (i.e., 45 compounds) were tested in an *in vitro* model of AD (i.e., monocytic cell lines, U-937, THP-1). It was shown that BDC stimulated the Vitamin D receptor (*VDR*) gene and had immunostimulatory effects such as regulating the transcription of genes implicated in innate immunity. VDR could act by protecting neurons from Aβ-induced toxicity and improving phagocytosis and Aβ degradation by promoting monocyte/macrophage maturation. Moreover, Liu et al. (2010) showed that curcumins may significantly suppress *BACE1* mRNA in cells from AD patients and confirmed the action of curcumins on gene expression.

The aim of the present work was to evaluate the protective effects of curcuminoids in blood cells from AD patients. Accordingly, we investigated the effect of several curcuminoids (Gagliardi et al., 2012) on NF-κB gene expression and compared the result to the clearance of Aβ in cells from AD patients. Numerous papers have reported effects of curcuminoids on cellular lines and animal models (Gagliardi et al., 2012), but few reports are available about their effect on primary cells from AD patients. To address this, we investigated the efficacy of curcuminoids on peripheral blood mononuclear cells (PBMCs) from AD patients and compared the results with matched control cells. Human PBMCs appear to be a good model system to study neurodegenerative processes because they have been shown to share much of the non-synaptic biochemical environment of neurons as well as signaling pathways of the (CNS’s) immune cells (Gagliardi et al., 2013; Arosio et al., 2014). In this report, based on results from *in vitro* cell-based studies with PBMCs, we describe the identification of a potent curcuminoid that modulates neurodegenerative signaling pathways *in vitro*. The results may be of relevance for future clinical studies aimed at increasing the clearance of Aβ and improving cognitive impairment.

## Materials and Methods

### AD Patients and Healthy Volunteers

Clinical and neurological examinations of AD patients were conducted at the IRCCS Mondino Foundation (Pavia, Italy). Healthy volunteers were recruited to the Immunohematological and Transfusional Service and Centre of Transplantation Immunology (IRCCS Foundation “San Matteo,” Pavia, Italy). Peripheral blood samples were collected from 30 AD patients with no family history of dementia and 28 healthy volunteers over 65 years of age after obtaining written informed consent (Table 1). Before being enrolled, the subjects that participated in the study signed an informed consent form (Protocol n°375/04 – version 07/01/2004). AD diagnosis was made according to the National Institute of Neurological and Communicative Disorders and Stroke and the AD and Related Disorders Association (NINCDS-ADRDA) criteria (Aggarwal and Harikumar, 2009). Quantification of neuropsychological testing, a detailed interview and clinical examinations were conducted with each subject. The severity of dementia in the AD patients was evaluated by the Mini Mental State Examination (MMSE) and carried out in accordance with The Code of Ethics of the World Medical Association (Declaration of Helsinki).

**Table 1:** Characteristics of subjects recruited for this study.

	AD	CTRs
	(*n* = 30)	(*n* = 28)
Age (M ± SD)	76.3 ± 4.3	75.7 ± 7.4
**Sex**		
Males n (%)	16 (53,3%)	12 (42,8%)
Females n (%)	14 (46.7%)	16 (57,2%)


*Values are reported as average ± standard deviation. The percentage of male and female subjects is also indicated.*

### PBMC Isolation and Treatment

**FIGURE 1:**
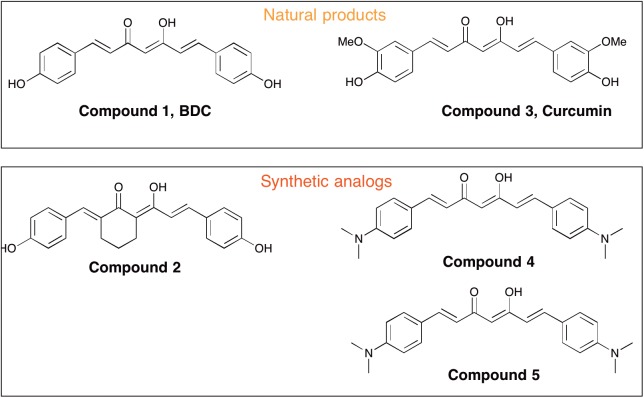
Chemical structures of curcuminoids used for cell treatment.

**FIGURE 2:**
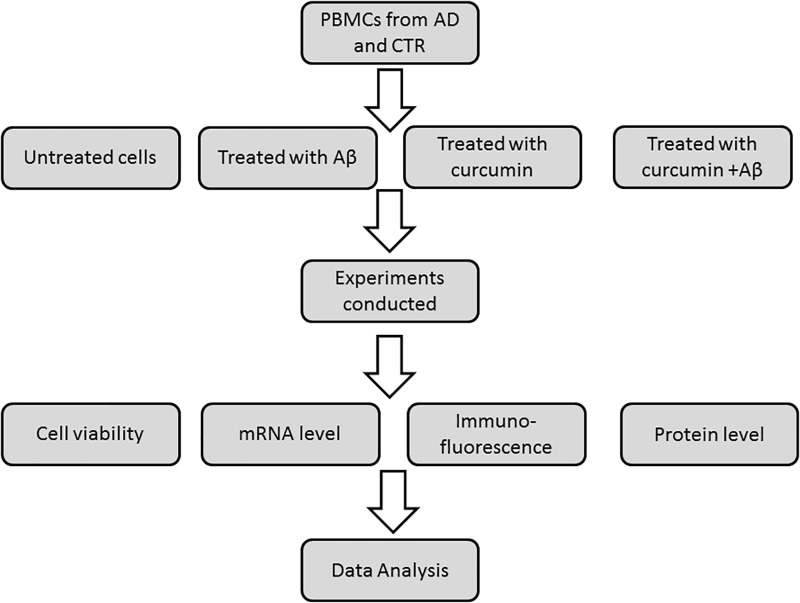
Schematic representation of experimental plan.

Peripheral blood mononuclear cells were prepared by layering peripheral blood on Ficoll-Histopaque (density = 1.077) and centrifugation at 950 ×*g* for 30 min. After isolation on a Ficoll-Histopaque layer (Sigma, Italy), cell viability was assayed by a trypan blue exclusion test and by cytometric analysis (Strober, 2001). Viable cells were used for *in vitro* studies with curcumins. PBMCs (5 × 10^6^ cells with viability ≥80%) were independently treated for 24 h with five different curcumins (0.1 μM) obtained from the Human BioMolecular Research Institute in San Diego, CA, United States. The curcumins were placed in DMSO and administered to cells in media. The five curcuminoids with the greatest potency for inducing expression of genes relevant to Aβ phagocytosis were selected based on results from a previous report (Gagliardi et al., 2012). The chemical structures of curcumins used in the studies are shown in Figure 1. About 5 × 10^6^ PBMCs were treated for 24 h with 0.1 μM of each curcuminoid and then with Aβ 1–42 (1 μM) (Sigma-Aldrich) for an additional 24 h. The conditions for tests included (Figure 2): untreated, treated only with curcuminoids, treated only with Aβ and, finally, treated with both curcuminoids and Aβ.

### Cell Viability

Trypan blue exclusion tests were conducted to assay cell viability after treatments. Curcuminoids and Aβ at 0.001–10 μM were used to treat PBMCs from AD patients and healthy volunteers for 24 h. The concentration range was determined based on data from similar studies carried out in different experimental models (Gagliardi et al., 2012).

### High-Performance Liquid Chromatography (HPLC)

To confirm the presence/absence of intact curcuminoids in cell culture supernatants and cell pellets, an HPLC method with ultraviolet (UV) detection for the determination of the selected compounds was used. A 1000 μL aliquot of cell culture supernatant was introduced onto an EMPORE Extraction Disk Cartridge (Sigma-Aldrich) under vacuum at 15 in Hg. Cartridges were conditioned sequentially with 1000 μL of methanol and 1000 μL of water. Interfering substances were washed out with water (1000 μL). Analytes were eluted with acetonitrile (80 μL) and water (120 μL). The eluate was then centrifuged at 1500 ×*g* for 5 min at 4°C and 50 μL was injected onto the HPLC system. HPLC analyses were carried out using a Shimadzu LC-10Av chromatograph (Shimadzu Scientific Instrument, Inc., Columbia, MD, United States) equipped with a System Controller SCL-10Avp, an LP-10ADvp pump, an on-line Degasser DGU-14A, a SIL-10ADvp auto injector and a model CTOXX temperature controller. The system was connected to a LaChrom L-7400 variable wavelength detector (Merck, Darmstadt, Germany). Separation of selected compounds was achieved on a Chromolith Performance Column (100 mm × 4.6 mm i.d., RP-18e, Merck, Darmstadt, Germany), protected by a guard column of the same composition (5-4.6 mm i.d., RP-18e, Merck, Darmstadt, Germany). The mobile phase was prepared by adding 1 mL of 85% phosphoric acid to 1 L of a mixture of water/acetonitrile (60:40 vol/vol) and thoroughly mixed. The column temperature was maintained at 50°C and the flow rate was 1.5 mL/min. Curcuminoids were detected at 420 nm. Data were acquired and analyzed using the Shimadzu LabSolution Lite software.

### RNA-Seq

RNA was extracted using the Maxwell 16, Blood kit (Promega and automated purification system Maxwell 16). RNA was analyzed by a Bioanalyzer spectrophotometer to verify the quality and the concentration and stored at -80°C. RNA libraries were prepared by TruSeq Targeted RNA Expression NF-κB Panel (Illumina, United States) according to the manufacturers’ instructions. The panel was integrated by BACE1 as an AD-linked gene, and VDR and MGAT3 to confirm the involvement of these two genes along the lines reported previously (Gagliardi et al., 2012). RNA processing and RNA-seq analysis was carried out using Illumina MiSeq sequencer. RNA sequencing data is available in GEO repository (GSE122438).

### Bioinformatics Analysis

Differential gene expression analysis was conducted on data obtained with TruSeq Targeted RNA Expression Kit with a MiSeq sequencer. Cells from AD patients and healthy volunteers were treated with different curcuminoids, both separately and in combination and were compared to identify differentially expressed genes.

The tables of counts obtained via MiSeq reporter analysis was imported in R^1^ and differentially expressed genes were evaluated using a negative binomial GLM approach (EdgeR Bioconductor). Batch effects were removed with the RemoveBatchEffects function. Differentially expressed genes had | log2FC | > 1 and *p*-values <0.1.

To validate the expression profiles obtained by RNA-Seq, real-time PCR was conducted on *MAGT3*, *VDR*, *NF-κB*, and *BACE1* genes selected for high or low expression levels.

### Immunofluorescence Microscopy

1 × 10^5^ cells were placed on a poly-L-lysine slide (Thermo Fisher Scientific) and incubated at 37°C to allow cell attachment to the slide. Cells were rinsed with 1× PBS and fixed in 4% paraformaldehyde in 1× PBS. Fixed cells were washed with 1× PBS and blocked with 5% normal goat serum in 0.1% Tween-PBS for 1 h. Incubation with the primary mouse anti-beta amyloid antibody (1:100 dilution; Abcam) was conducted in blocking solution overnight at 4°C. The fluorescently tagged secondary antibody CFTM 488A goat anti-rabbit (1:700 dilution; Sigma-Aldrich) was used for detection. Slides were mounted with Prolong^®^ Gold anti-fade reagent with 4′6-diamidino-2-phenylindole (DAPI) (Invitrogen) and images were acquired by confocal microscopy (Leica DM IRBE, Leica Microsystems Srl, Italy).

### AlphaLISA

Immediately after PBMCs were treated with curcumins, they were centrifuged at 16,000 rpm for 8 min at room temperature and the cell pellet was incubated with lysis buffer supplemented with protease inhibitors at 4°C for 10 min and then centrifuged at 18,000 ×*g* for 15 min at 4°C. The supernatant was used for measuring Aβ 1–42. The Aβ 1–42 content was determined using the AlphaLISA technique with the AlphaLISA human amyloid beta 1–42 (high specificity) kit (PerkinElmer) according to the manufacturers’ instructions. In the assay, biotinylated antibody against Aβ 1–42 and Acceptor beads (PerkinElmer) conjugated to a 2nd antibody against Aβ 1–42 was added to samples in 1× HiBlock AlphaLISA buffer on the assay plate. One antibody was specific to the β-secretase cleavage site at the N-terminus (mouse monoclonal antibody, clone number 82E1), the second antibody was specific to the C-terminus (mouse monoclonal antibody, clone number 12F4). The plate was sealed and incubated for 1 h at room temperature. Streptavidin-coated Donor beads were then added under subdued lighting, followed by 1 h incubation in the dark to bind the biotinylated antibody. When both antibodies bound Aβ 1–42 fragments, they were in close enough proximity and the excitation at 680 nm resulted in the production of ^1^O_2_ from the Donor beads. Energy is transferred from the singlet oxygen to the Acceptor beads, subsequently culminating in light production at 615 nm that was detected using an EnVision plate reader (PerkinElmer). For each group of cells, Aβ 1–42 content was determined by interpolating the standard curve using 4PL non-linear regression. As a control the same procedure was conducted with PBMCs treated only with Aβ in the absence of BDC.

## Results

### Cell Viability

**FIGURE 3:**
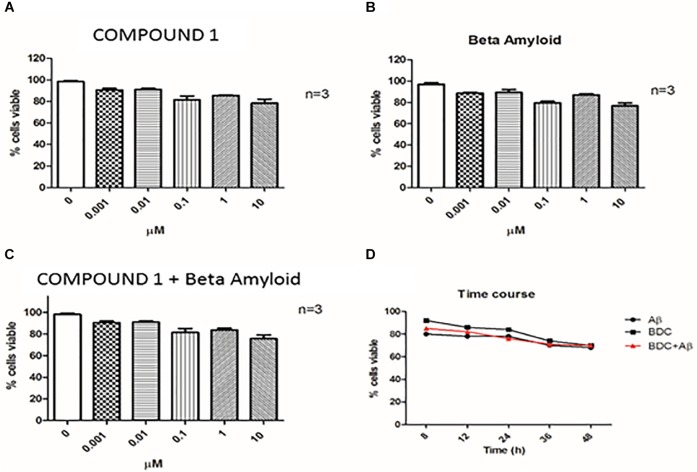
Cell viability. Cell viability was assessed by Trypan Blue exclusion test. PBMCs (Peripheral blood mononuclear cells) were treated with COMPOUND 1 at 0.001–10 μM **(A)**, with amyloid beta **(B)**, with both COMPOUND 1 and amyloid beta **(C)**, and the Time Course for all three treatments **(D)**.

Cells treated with 10 μM curcumin and 10 μM Aβ showed a greater decrease in viability compared with lower concentrations examined. Therefore, curcumins at 0.1 μM and Aβ at 1 μM were used for experiments (Figures 3A,B). Treatments with both molecules at the chosen concentrations did not show a significant alteration of cells viability (Figure 3C).

### Analysis of Curcuminoids in Supernatants and Cellular Pellets

**FIGURE 4:**
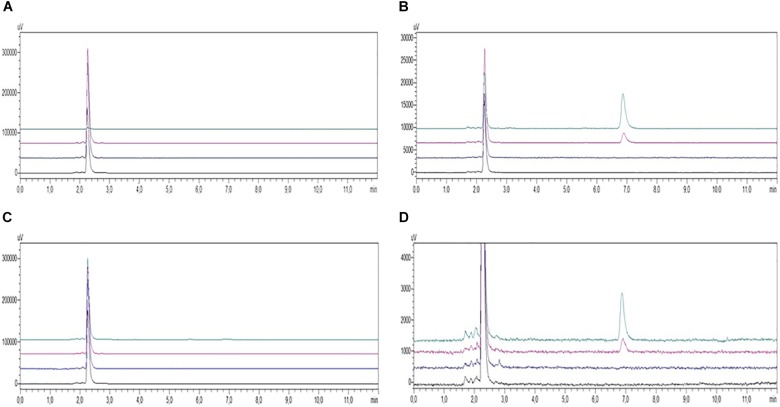
HPLC-UV chromatograms of PBMC samples from AD patients (**A**, supernatants; **B**, pellets) and healthy controls (**C**, supernatants; **D**, pellets) incubated in the absence (black, untreated; blue, treated with Aβ) or presence (red, treated with COMPOUND 1 and Aβ; green, treated with COMPOUND 1). COMPOUND 1 10 μM for 24 h at 37°C in cellular pellets and supernatants.

Although 10 μM curcuminoid was the concentration with the greatest impact on cells viability, the percentage of apoptosis was considered acceptable (20%) so it was used for HPLC analysis due to the sensitivity of the method. Under the chromatographic conditions the duration of each chromatographic run was 8 min and COMPOUND 1 eluted at 7 min. HPLC analysis conducted on PBMC samples from AD patients and healthy volunteers treated with curcuminoids (i.e., 10 μM for 24 h) showed the presence of compounds in the cellular pellet. Curcumins were absent in the supernatant of cells from both AD patients and healthy subjects treated with curcumins (Figures 4A,C). The presence of curcuminoids in the cellular pellet was reasonable based on the lipophilicity and protein binding properties of the compounds and confirmed that they partitioned into cells (Figures 4B,D).

### mRNA Expression Profiles in PBMC Samples From AD Patients and Healthy Volunteers

Compared to untreated PBMC cells of AD patients, mRNA expression of 12 selected genes in PBMCs from healthy controls showed a significant difference (Table 2). Among the 12 genes examined, 6 genes (i.e., *NF-κB*, *BACE1*, *VDR*, *MGAT3*, *TLR7*, *and TLR8*) were previously reported to be targets of curcumin action. Alterations of *NF-κB*, *BACE1*, *MGAT3*, and *VDR* gene expression were validated with Real-Time PCR. The results confirmed the next generation sequencing (NGS) data that showed an increase of *NF-κB* and *BACE1* in cells from AD patients compared to cells from healthy volunteers and a decrease of *MGAT3* and *VDR* in cells from AD patients compared to cells from healthy controls (Table 2).

**Table 2:** Statistically significant differentially expressed mRNAs in PBMCs from AD patients, compared to controls.

Genes	logFC	FDR
IFNA1	–5,068413935	0,000490531
TNFSF14	–3,658110471	0,000214144
IL1RN	–3,640381192	1,66533E-05
ICAM1	–3,517928503	2,5152E-10
RELB	–3,095303227	0,000234517
IRAK1	–3,029891347	4,04098E-06
TRADD	–2,854373904	0,000398196
LTBR	–2,307780352	2,51562E-06
NFKB2	–2,264966319	0,000398196
IRAK2	–2,200464564	0,000137778
PLAU	–2,199648435	0,007553911
VCAM1	–2,155015236	0,016657456
TICAM1	–1,920135409	0,005236392
NFKBIE	–1,719026115	0,000234517
MMP9	–1,672886236	0,01143092
TLR9	–1,622129773	0,004608774
TNFRSF1A	–1,620666312	0,001389893
TNFAIP3	–1,538997066	0,004768896
IKBKG	–1,53268413	0,003992276
CXCL1	–1,397816804	0,081832301
TNFRSF10B	–1,312723196	0,000398196
GADD45B	–1,311377024	0,006364934
SOD2	–1,268237434	0,005538847
VDR	–1,25733448	0,001665746
ELK1	–1,025052967	0,016657456
MGAT3	–0,99923567	0,006115305
XIAP	–0,997730728	0,061153047
EGR1	–0,890765445	0,055696396
TLR6	–0,760645822	0,04190734
TNFRSF1B	–0,518149337	0,081953512
REL	1,079234282	0,077638505
MYC	1,20491787	0,077638505
CD69	1,482195001	0,027318454
BIRC3	1,494054357	0,01156061
CD27	1,53452282	0,046143966
MALT1	1,882689476	0,022264388
CHUK	2,176330259	0,000748631
BACE1	2,25413641	0,068195351
TLR3	2,995531758	0,000234517
NFKB	3,002989135	0,005755391


*Transcripts were considered as differentially expressed when | log_2_(disease sample/healthy control) |≥ 1 and a FDR ≤ 0.1.*

### Treatment of PBMCs From AD and Healthy Volunteers With Curcuminoids

Peripheral blood mononuclear cells from AD patients and healthy volunteers treated with curcuminoids (i.e., 0.1 μM, 24 h) showed a significant alteration in gene expression in cells from AD patients (Supplementary Table S1). Compared to cells from healthy volunteers, COMPOUND 1 was the most potent compound in regulating genes expression and caused a twofold increase after treatment in cells from AD patients Compared to PBMCs from healthy controls, down-regulation of *MGAT3* was observed (log2FC = 18) in untreated PBMCs of AD patients. In contrast, over-expression of *MGAT3* was observed in PBMCs of AD patients treated with curcuminoids (i.e., 7- and 18-fold increase with COMPOUND 1 and 3, respectively). This showed that curcuminoids were able to induce transcription of *MGAT3* in PBMCs from AD patients even in the absence of Aβ.

### Treatment of PBMCs From AD and Healthy Volunteers With Aβ

Treatment of PBMCs from AD patients with Aβ 1–42 (1 μM) caused significant alterations in genes expression but only in cells from AD patients (Supplementary Table S2). Compared to untreated PBMC cells from healthy volunteers, results showed that treatment of PBMCs from AD patients afforded a greater number of altered genes. Compared to PBMCs from healthy volunteers, 20 genes were up-regulated in PBMCs from AD patients after treatment with Aβ (0.1 μM, 24 h). For example, *MGAT3* and *VDR* were increased log2FC, five and twofold, respectively. TLR4, 5, 7, and 8 were up-regulated log2FC, X-, X-, etc-fold, respectively. Compared to PBMCs from healthy volunteers, 15 genes were down-regulated in PBMCs from AD patients treated with 0.1 μM for 24 h. For example, *CCR5*, *CD27*, and *CD83* genes involved in inflammatory pathway were down-regulated four-, three-, and twofold log2FC, respectively.

### Effect of COMPOUND 1 Treatment on PBMCs From AD Patients and Healthy Volunteers

Individual PBMCs from 30 AD patients and 28 healthy volunteers were treated with COMPOUND 1 (i.e., 0.1 μM, 24 h), and then Aβ 1 μM was added to the cell culture for the next 24 h and Aβ and gene expression was quantified.

#### Aβ Protein Quantification

**FIGURE 5:**
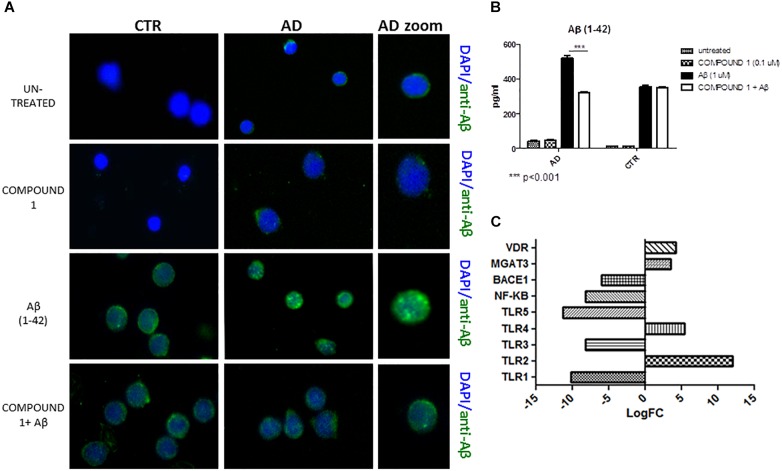
Peripheral blood mononuclear cells from AD patients and control subjects treated with Aβ (48 h) or untreated, COMPOUND 1 (24 h) and COMPOUND 1 (24 h) + Aβ (24 h). **(A)** Immunohistochemistry images; **(B)** Aβ Alpha Technology Assays; **(C)** Deregulated RNAs for PBMCs treated with Aβ and treated with COMPOUND 1 + Aβ.

Immunofluorescence results afforded a qualitative measure of Aβ in PBMCs. A decrease of green fluorescence in PBMCs from AD patients in the presence of COMPOUND 1 (0.1 μM, 24 h) showed a decrease of Aβ (Figure 5A).

An AlphaLISA assay showed a quantitatively and statistically significant decrease (*p* < 0.01) of Aβ in PBMCs from AD patients treated with COMPOUND 1 (Figure 5B). In contrast, a statistically significant difference was not observed in PBMCs from healthy volunteers treated with COMPOUND 1 (0.1 μM, 24 h).

#### Gene Expression

**FIGURE 6:**
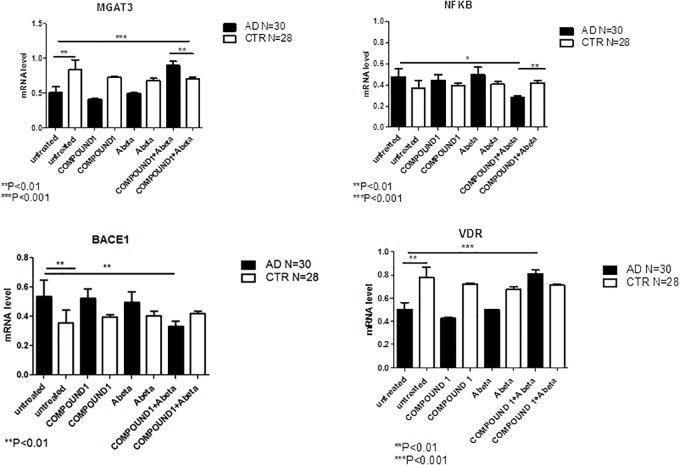
*MGAT3*, *NF-κB*, *BACE1*, and *VDR* gene expression in PBMCs from AD patients and control subjects treated with Aβ (48 h) or untreated, and COMPOUND 1 (24 h) and COMPOUND 1 (24 h) + Aβ (24 h).

NGS data showed that, compared to PBMCs from healthy volunteers, treatment of PBMCs from AD patients with COMPOUND 1 (i.e., 0.1 μM, 24 h) caused transcriptional changes in 9 genes (Figure 5C). Compared to cells from healthy volunteers *TRL2* and *TRL4* were up-regulated (5- and 10-fold, respectively) after treating PBMCs from AD patients with COMPOUND 1 (0.1 μM, 24 h) based on quantitative RT-PCR analysis. Compared to PBMCs from healthy volunteers, treatment of PBMCs from AD patients with COMPOUND 1 (0.1 μM, 24 h) showed a statistically significant increase (*p* < 0.01) of genes involved in Aβ clearance (i.e., *MGAT3* and *VDR*, four and fivefold increase, respectively) and a decrease in *NF-κB* and *BACE1* (6- and 8-fold-decrease, respectively) (Figure 6).

### Effect of COMPOUND 2 Treatment on PBMCs From AD Patients and Healthy Volunteers

Peripheral blood mononuclear cells from 30 AD patients and 28 healthy subjects were treated with COMPOUND 2 (i.e., 0.1 μM for 24 h) and then Aβ (i.e., 1 μM) was added to the cells for the next 24 h.

#### Aβ Protein Quantification

**FIGURE 7:**
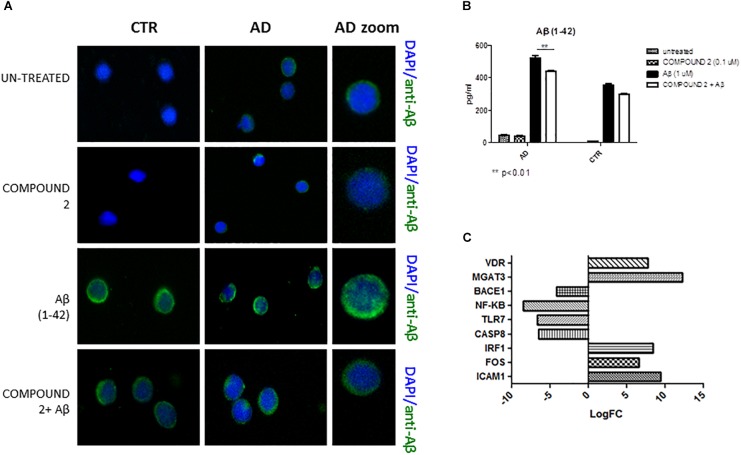
Peripheral blood mononuclear cells from AD patients and control subjects treated with Aβ (48 h) and untreated, COMPOUND 2 (24 h) and COMPOUND 2 (24 h) + Aβ (24 h). **(A)** Immunohistochemistry images; **(B)** Aβ Alpha Technology Assays; **(C)** Deregulated RNAs between PBMCs treated with Aβ and treated with COMPOUND 2 + Aβ.

Compared to healthy volunteers, qualitative results of immunofluorescence and quantitative results of AlphaLISA showed a significant decrease of Aβ (*p* < 0.01) in PBMCs from AD patients treated with COMPOUND 2 (0.1 μM, 24 h) (Figures 7A,B). No significant differences in Aβ was observed for PBMCs from healthy volunteers treated with COMPOUND 2 although there was a slight reduction in Aβ deposits.

#### Gene Expression

**FIGURE 8:**
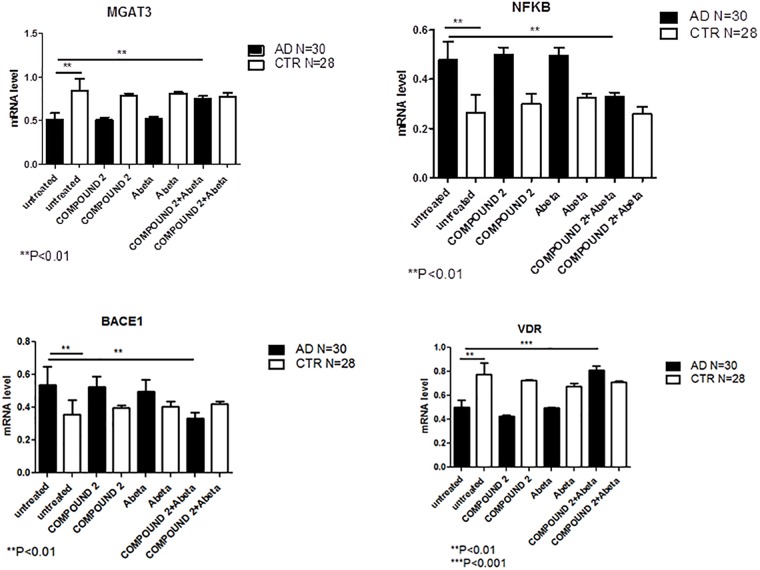
*MGAT3*, *NF-κB*, *BACE1*, and *VDR* gene expression in PBMCs from AD patients and control subjects treated with Aβ (48 h) or untreated, and COMPOUND 2 (24 h) or COMPOUND 2 (24 h) + Aβ (24 h).

Compared to healthy volunteers, treatment of PBMCs from AD patients with COMPOUND 2 (i.e., 0.1 μM for 24 h) showed transcriptional changes in 9 genes (i.e., *ICAM1*, *FOS*, *IRF1*, *MGAT3*, *VDR*, *TLR7*, *CASP8*, *NFKB*, and *BACE1*, 9-, 6-, 8-, 12-, and 7-fold, increase, respectively, and 6-, 6-, 8-, and 4-fold, decrease, respectively) (Figure 7C). Data from quantitative RT-PCR were in accordance with NGS data described above and with COMPOUND 2 treatment results that showed a decrease in *NF-κB* and *BACE1* and an increase of *MGAT3* and *VDR* (*p* < 0.01) (Figure 8).

### Effect of COMPOUNDS 3–5 Treatment on PBMCs From AD Patients and Healthy Subjects

Peripheral blood mononuclear cells from 30 AD patients and 28 healthy volunteers were treated with COMPOUND 3 (i.e., 0.1 μM for 24 h) and then Aβ (i.e., 1 μM) was added to cells for the next 24 h.

#### Aβ Protein Quantification

**FIGURE 9:**
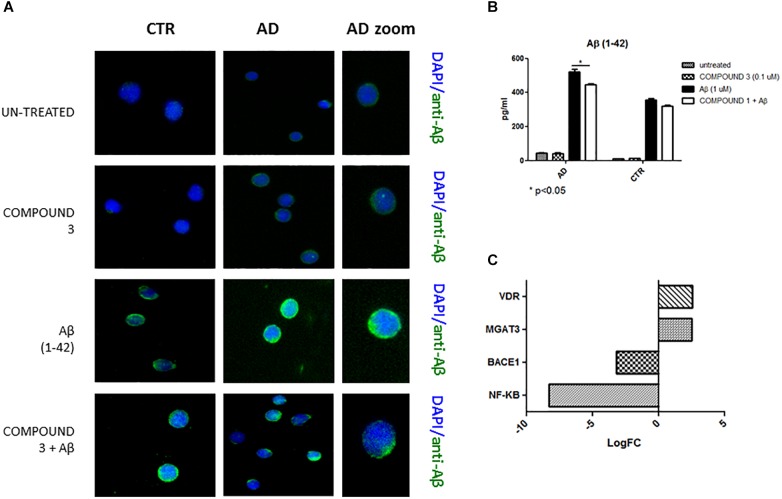
Peripheral blood mononuclear cells from AD patients and control subjects treated with Aβ (48 h) or untreated, COMPOUND 3 (24 h) or COMPOUND 3 (24 h) + Aβ (24 h).

Qualitative results from immunofluorescence showed a decrease in Aβ similar to COMPOUNDS 1 and 2 (Figure 9A), but quantitative results from AlphaLISA were less significant (*p* < 0.05) than the results of COMPOUNDS 1 and 2 treatments (Figure 9B). No significant differences in immunofluorescence were observed in PBMCs from healthy subjects treated with COMPOUND 4 and 5, despite evidence there was a slight decrease in Aβ deposits.

#### Gene Expression

**FIGURE 10:**
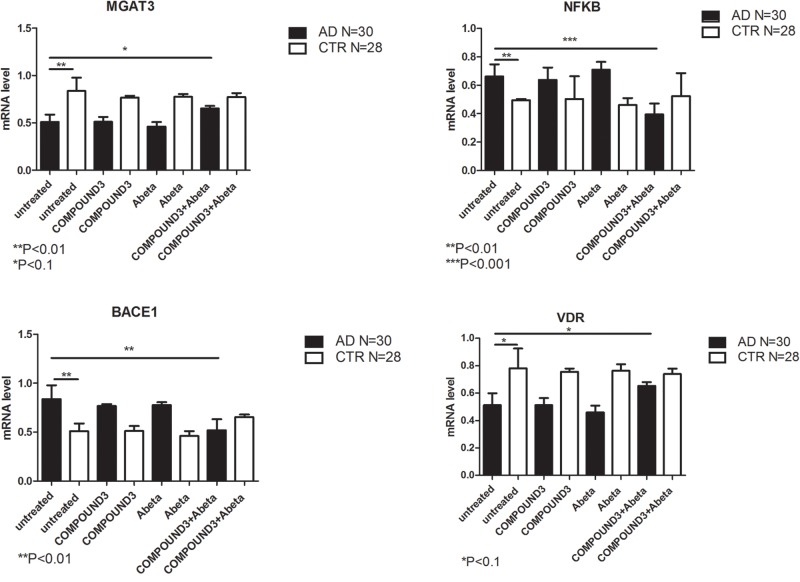
*MGAT3*, *NF-κB*, *BACE1*, and *VDR* gene expression in PBMCs from AD patients and control subject treated with Aβ (48 h) and untreated, COMPOUND 3 (24 h) or COMPOUND 3 (24 h) + Aβ (24 h).

COMPOUND 3 decreased gene expression of target genes compared to COMPOUND 1 or COMPOUND 2. NGS analysis revealed alteration in 4 genes (i.e., *MGAT3*, *CD40*, *TLR8*, and *PTGS2*) (Figure 9C). Validation of NGS results with quantitative RT-PCR confirmed a statistically significant (*p* < 0.01, *p* < 0.001) increase of *MGAT3* and *VDR* and a decrease of *NF-κB* and *BACE1* (Figure 10).

## Discussion

Alzheimer’s Disease is a progressive, degenerative brain disease that is the most common cause of dementia in elderly people. One of the main pathogenic factors in AD includes amyloid plaques derived from cleavage of amyloid precursor protein (APP) by BACE1 in neurons. Moreover, it has been shown that regulation of gene expression related to Aβ clearance is also altered in AD (Fiala et al., 2007; Sebollela et al., 2012).

The strength of this work is the use of human PBMCs from AD and age-matched control individuals to examine curcuminoid effects on Aβ clearance gene regulation as anti-AD compounds. We analyzed the protective effect of curcuminoids and investigated two main pathways: BACE1/NF-κB and MGAT3/VDR. The results of the studies may be relevant to the use of curcuminoids in prevention of AD working via Aβ clearance mechanisms. In particular, we studied how curcumins act on cell susceptibility to Aβ accumulation. We conducted studies that showed treatment with curcumin that may decrease the predisposition of Aβ to accumulate into cells.

In untreated PBMCs, our data showed that, *BACE1* and *NF-κB* genes were up-regulated in PBMCs from AD patients compared to PBMCs from healthy volunteers. Increases in both *BACE1* and *NF-κB* have been reported in AD brain (Chen et al., 2012; Sebollela et al., 2012) but have not been described in peripheral cells of AD patients. Chen and collaborators showed that *NF-κB* regulates *BACE1* with consequent toxic Aβ generation (Chami et al., 2012). Data herein showed an increase of Aβ 1–42 in PBMCs from AD patients where corresponding elevated levels of *NF-κB* and *BACE1* mRNAs were present. Recently, a *NF-κB*-binding element was identified in the human *BACE1* promoter region (Chami et al., 2012).

*MGAT3* and *BACE1* working together may also be involved in the Aβ clearance pathway: MGAT3 may regulate the degradation of BACE1 in lysosomes (Kizuka et al., 2016). This constitutes one important step related to Aβ-plaque formation and MGAT3 may also stabilize BACE1 in oxidative stress conditions (Hickman et al., 2008; Palmigiano et al., 2016). Compared to PBMCs from controls, *MGAT3* and *VDR* have been reported to be down-regulated in PBMCs from AD subjects (Fiala et al., 2007). In addition, compared to healthy subjects, macrophage function is decreased in subjects with AD (Hickman et al., 2008). An increase of MGAT3 functional activity may improve macrophage viability, because it has been previously shown that MGAT3 silencing inhibited Aβ phagocytic function of control monocytes.

To support the hypothesis that curcuminoids selectively enhance Aβ phagocytosis, attenuate APP maturation and alter gene transcription in PBMCs of AD patients we tested the effects of curcuminoids on PBMCs from patients with AD disease (Farmer et al., 2000; Cashman et al., 2012). In brief, our data shows curcumin treatment (i.e., particularly BDC) produced a decrease of intracellular Aβ aggregates, an increase of phagocytosis (e.g., the MGAT3 pathway) and a reduction of inflammation.

Results of treatment of PBMCs from AD patients with curcuminoids (and subsequently treated with Aβ to induce Aβ aggregation) showed an ability to act on cell susceptibility to Aβ accumulation for three curcumin analogs (COMPOUNDS 1, 2, and 3). COMPOUND 1 appeared to be the most potent.

Data herein showed *NF-κB* and *BACE1* were decreased after pre- with curcuminoids. Because *NF-κB* signaling leads to an up-regulation of *BACE1* gene expression and facilitates APP processing in PBMCs from AD patients, its decline following treatment would decrease the formation of Aβ fragments by BACE1. In addition, the decrease in *NF-κB* leads to suppression of the inflammatory cascade, one of the major pathways in AD. PBMCs from AD patients treated with curcumin led to an increase in gene expression of *MGAT3*, *VDR*, and *TLR4* similar to controls. Normal elevated levels of *MGAT3* have been associated with an effective degradation and disposal process of Aβ, although the detailed mechanism has not yet been fully elucidated (Kizuka et al., 2015).

Vitamin D3, VDR’s endogenous ligand, regulates transcription of genes of innate immunity (Lin, 2016). It has been hypothesized that Vitamin D3 possesses immunostimulant effects and also induces phagocytosis and degradation of Aβ by monocytes/macrophages maturation (Aggarwal and Harikumar, 2009; Masoumi et al., 2009). We suggest that increased MGAT3 and VDR inhibit Aβ accumulation and decreases inflammation. Moreover, in most systems, vitamin D inhibits the activation of NF-κB (Chen et al., 2013). VDR stimulation induced by curcuminoids could shift this balance to and increase inhibitory activity of VDR on NF-κB (Geldmeyer-Hilt et al., 2011). For both MGAT3 and VDR pathways, an increase of TLR4 may also play a role in NF-κB repression and on macrophage activation even if it still remains unclear what acts as a TLR4 activator.

Future work will be to identify and characterize molecular factors of the inflammatory process and the relationships between them. Our data support continued pharmacological studies. In the future, curcumins may be established as important dietary supplements in asymptomatic AD subjects (Reddy et al., 2018). Finally, BDC may be useful in subjects that have more propensities to develop AD or in subjects that are in the early stages of Aβ accumulation but that is still not damaging to prevent the degeneration to Aβ plaques, a key event in AD pathogenesis.

## Conclusion

The data presented herein may contribute to the understanding of the etiopathogenetic mechanism of AD. Further studies may be warranted to investigate a possible preventive approach in human subjects that may be susceptible to develop AD but that still do not have clinical signs. In a cell model, we have shown that Treatment with curcuminoids inhibits the pathological mechanisms that are responsible for some AD pathways such as the activation of inflammation and of accumulation of Aβ.

## Author Contributions

SG, VF, and CP ran the experiments and wrote the manuscript. SS ran the experiments. SZ performed the bioinformatic analysis. PR performed the HPLC analysis. SB recruited the healthy subjects. AC and ES recruited the patients. OP and CC revised the paper. JC provided the curcumins and revised the paper.

## Conflict of Interest Statement

The authors declare that the research was conducted in the absence of any commercial or financial relationships that could be construed as a potential conflict of interest. The reviewer AA and handling Editor declared their shared affiliation at the time of review.

## Footnotes

1 https://www.r-project.org/
